# Application of an artificial neural network model for diagnosing type 2 diabetes mellitus and determining the relative importance of risk factors

**DOI:** 10.4178/epih.e2018007

**Published:** 2018-03-10

**Authors:** Shiva Borzouei, Ali Reza Soltanian

**Affiliations:** 1 Department of Endocrinology, School of Medicine, Hamadan University of Medical Sciences, Hamadan, Iran; 2 Department of Biostatistics, School of Public Health, Hamadan University of Medical Sciences, Hamadan, Iran; 3 Modeling of Noncommunicable Diseases Research Center, School of Public Health, Hamadan University of Medical Sciences, Hamadan, Iran

**Keywords:** Statistical model, Glycated hemoglobin A, Epidemiology, Iran

## Abstract

**OBJECTIVES:**

To identify the most important demographic risk factors for a diagnosis of type 2 diabetes mellitus (T2DM) using a neural network model.

**METHODS:**

This study was conducted on a sample of 234 individuals, in whom T2DM was diagnosed using hemoglobin A1c levels. A multilayer perceptron artificial neural network was used to identify demographic risk factors for T2DM and their importance. The DeLong method was used to compare the models by fitting in sequential steps.

**RESULTS:**

Variables found to be significant at a level of p<0.2 in a univariate logistic regression analysis (age, hypertension, waist circumference, body mass index [BMI], sedentary lifestyle, smoking, vegetable consumption, family history of T2DM, stress, walking, fruit consumption, and sex) were entered into the model. After 7 stages of neural network modeling, only waist circumference (100.0%), age (78.5%), BMI (78.2%), hypertension (69.4%), stress (54.2%), smoking (49.3%), and a family history of T2DM (37.2%) were identified as predictors of the diagnosis of T2DM.

**CONCLUSIONS:**

In this study, waist circumference and age were the most important predictors of T2DM. Due to the sensitivity, specificity, and accuracy of the final model, it is suggested that these variables should be used for T2DM risk assessment in screening tests.

## INTRODUCTION

Type 2 diabetes mellitus (T2DM) is a non-contagious and chronic disease [1]. T2DM can cause many other diseases, such as cardiovascular disease [2], stroke [3], blindness [4], and loss of renal function [5].

The prevalence of diabetes is increasing. Worldwide, 285 million people had diabetes in 2010, compared to 422 million in 2014 [6] and this number is projected to increase to 438 million in 2030 [7] and 592 million in 2035 [8]. The prevalence of diabetes in low-income or moderate-income countries is higher than in high-income countries [7], and it accounts for a large share of the mortality and disability rate in such communities [6]. One of the reasons for the high prevalence of diabetes in low-income countries may be low levels of knowledge and awareness about diabetes [9].

In 2010 and 2012, the number of undiagnosed cases of diabetes was reported to be 7 and 1.8 million, respectively, corresponding to approximately a quarter of the diagnosed cases [8]; it is also important to note that the cost of treating diabetes is greater than that of prevention.

Therefore, the prevention of diabetes mellitus is of high importance in all communities. The first step in the prevention of T2DM is to identify its risk factors. Our literature review showed that factors such as age [10,11], sex [10,12], family history of diabetes [11, 13], hypertension [14], obesity [10,15], abdominal obesity [16], stress in the workplace or home [17,18], a sedentary lifestyle [19,20], smoking [21], insufficient fruit and vegetable consumption [22], and physical activity [23,24] are risk factors associated with T2DM.

Many previous studies have predicted T2DM based on individuals’ lipid profile (e.g., low-density lipoprotein cholesterol, high-density lipoprotein cholesterol, fasting blood sugar, etc.) [1,8,25]. Because such variables are costly to measure, we instead used variables that do not require much cost to measure them (e.g., sex, age, body mass index [BMI], etc.).

Inadequate healthcare facilities in many countries, especially low-income countries, as well as the complete failure to prevent T2DM, spurred us to identify the importance of various demographic risk factors for T2DM. Artificial neural networks (ANNs) are an advanced method for estimating outcomes and prioritizing risk factors. The two medical criteria for diagnosing T2DM (fasting blood sugar and hemoglobin A1c [HbA1c]) may not be cost-effective for T2DM screening on the community level.

The ANN technique is an advanced modeling technique based on brain neurons that has been widely used in recent years, and can be helpful for diagnosing, estimating, and predicting various diseases [1].

Our aim is to present a diagnostic model that can predict and determine the importance of risk factors affecting T2DM using an ANN model.

## MATERIALS AND METHODS

### Setting and participants

This descriptive analytical study was conducted on a sample of 234 individuals referred to a diabetes center in the city of Hamadan (in western Iran) from November 27, 2015 to March 15, 2016. The Hamadan Diabetes Risk Score study enrolled 130 normal and 130 diabetic volunteers among individuals aged 18 or more who attended the Hamadan diabetes center as a patient’s companion. Of the volunteers without diabetes who were invited to participate (n= 130), only 106 had their HbA1c measured at the laboratory, whereas all individuals with diabetes did so.

The inclusion criteria for non-diabetic subjects were age ≥ 18 years; no mental disability; no history of type 1 diabetes, T2DM, or gestational diabetes; no current pregnancy (for female); and no current use of metformin or other glucose control drugs.

The inclusion criteria for the subjects with diabetes were age ≥ 18 years; no mental disability; the presence of T2DM without type 1 or gestational diabetes; and no current pregnancy (for female).

After obtaining informed consent, subjects were referred to the laboratory for HbA1c tests, and the diagnosis of subjects as having or not having diabetes was made based on the HbA1c results by an endocrinologist. We applied the American Diabetes Association criteria to the HbA1c results with cut-off points of less than 5.8% (< 40 mmol/mol) as normal, 5.8-6.4% (40-46 mmol/ mol) as pre-diabetes, and 6.5% and more (48 mmol/mol) as indicative of T2DM [26]. For better interpretation of the results, we divided the subjects into 2 groups: normal and diabetic (i.e., prediabetes+diabetes). Informed consent was obtained from all individual participants included in the study, and the ethical committee of Hamadan University of Medical Sciences approved the study (IR.UMSHA.REC.1394.238).

### Statistical analysis

Initially, using a univariate logistic regression analysis, we chose the risk factors that had a significance level of p<0.2 (Tables 1 and 2).

**Figure 1.**
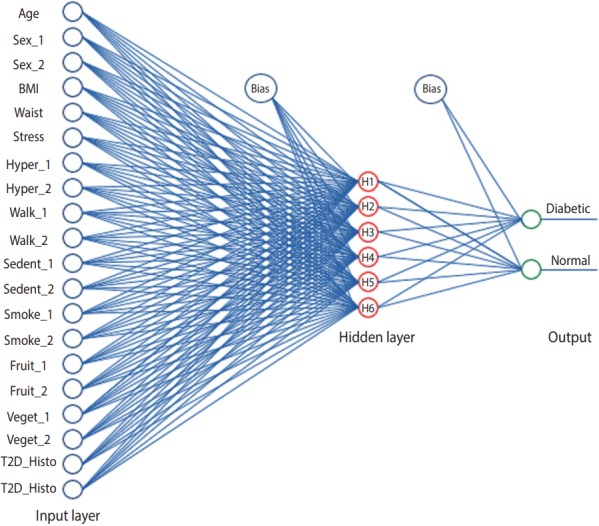
Artificial neural networks scheme of predictors of T2DM starting at the first step, with 20 inputs, 6 hidden layers (H1, ..., H6), and dichotomous output neurons. The encoded variables are presented in Table 1. BMI, Waist, Hyper_, Walk_, Sedent_, Veget_ and T2D_Histo denote body mass index; waist circumference, hypertension status, walking time, sedentary status, vegetables consumption and family history of type 2 diabetes mellitus, respectively.

In this study, we used a 3-layer ANN to model the risk factors of T2DM (Figure 1). The first layer considers input variables (i.e., neurons), the second layer considers hidden neurons, and the third considers the dichotomous output (diabetes status). The number of hidden layer neurons was determined by the rule proposed by Masters [27]. Therefore, for a 3-layer ANN with *p* input and *q* output neurons, the hidden layer would have $\sqrt{\left(p*q\right)}$ neurons [27].

The basic ANN was modeled as follows:

$$y_{i}\;=\;F\left[\sum_{i=1}^{p}w_{i}\chi_{i}\;+\;b_{i}\right]$$

where, *y_i_* denotes the output variables, *x_i_* (*i*= 1, 2, …, *p*) denotes the input variables, *w_i_* (*i*= 1, 2, …, *p*) denotes the optimum weights according to the input variables, and *p_i_* (*i*= 1, 2, …, *p*) denotes the bias term. In this study, the sigmoid function was used as an activation function.

To avoid overfitting, and to evaluate the model’s generalizability, the existing datasets were divided into 3 subsets for training (60.0%), testing (20.0%), and validation (20.0%) before the modeling process began [28]. In this study, an ANN multilayer perceptron with 3 layers and the Broyden-Fletcher-Golfarb-Shanno educational algorithm were used for modeling. The reason for choosing this algorithm was its high convergence rate compared to other algorithms.

Replication experiments were used to determine the number of hidden-layer neurons, the function of the layers, and the error function, so that at each stage 100 ANNs were modeled. In order to produce an appropriate model for predicting T2DM, in the first stage, all variables were considered in the model. In the second stage, the importance of risk factors was determined using the classification and regression tree strategy.

In the third stage, based on the backward method, less important risk factors for T2DM were eliminated. The modeling continued until the accuracy of the obtained models started to show significant differences from the first stage. Receiver operating characteristic (ROC) curves were used to compare the performance of the models. The DeLong method was used to compare the area under the ROC curve (AUC) before and after the removal of risk factors [29]. As shown in Table 1, we considered 15 features for each data sample. The diagnosis of T2DM by HbA1c was the output, and the other variables were inputs. Statistical software version R 3.2.2; neuralnet package (https://CRAN.R-project.org/package=neuralnet) was used to apply neural network modeling. To register individuals’ attributes, a form was used with 13 variables (Table 1).

## RESULTS

A total of 23 males (21.7%) and 83 females (78.3%), who had undiagnosed T2DM, participated in the study. The age range of the participants was 23-80 years old. Of the participants, 12.3% had hypertension, 46.2% walked for less than 30 min/d, and 50.0% reported often leading a sedentary lifestyle at work or at home. In the present study, 21 cases (19.8%) had a family history of T2DM, and 75 (70.1%) were non-smokers.

The risk factors were then entered into the multilayer perceptron ANN model. Sensitivity, specificity, and AUC were determined. The modeling was performed 6 times, with the following results for each step.

### First model

The important risk factors identified using the classification and regression tree were age (100.0%), hypertension (57.6%), waist circumference (55.5%), BMI (46.9%), a sedentary lifestyle (46.4%), smoking (41.7%), vegetable consumption (29.4%), family history of T2DM (27.0%), stress (21.3%), walking (18.3%), fruit consumption (8.0%), and sex (7.1%). The values in parentheses indicate the importance of the risk factors. Of all the risk factors, sex was the least important. Therefore, sex was eliminated from the model, and in the next step, the model was re-applied without sex.

### Second model

In this step, the multilayer perceptron ANN model without the variable of sex was implemented, with 11 risk factors. The importance of the risk factors in the new model was as follows: age (100.0%), waist circumference, (65.5%), stress (63.3%), BMI (63.3%), family history of T2DM (37.6%), vegetable consumption (31.8%), smoking (31.5%), a sedentary lifestyle (29.8%), hypertension (28.6%), walking (18.9%), and fruit consumption (11.1%). The DeLong method showed that the AUC of the second model did not show a significant difference (p= 0.841) compared to the first model. Therefore, the modeling process continued.

### Third model

After removing the fruit consumption variable as the least important risk factor, a multilayer perceptron ANN model with 10 variables was executed. The importance of the risk factors in the model was as follows: age (100.0%), waist circumference (54.4%), BMI (35.2%), family history of T2DM (33.3%), hypertension (28.8%), smoking (25.1%), stress (19.8%), vegetable consumption (18.9%), a sedentary lifestyle (18.1%), and walking (4.9%). The DeLong method found that the AUC of the first and the third model did not show a significant difference (p= 0.735). Therefore, we removed walking as the risk factor with the least importance from the model.

### Fourth model

After removing the walking variable as the least important risk factor in the previous model, a multilayer perceptron model was executed with the 9 remaining risk factors. The importance of the risk factors in the model was as follows: age (100.0%), stress (77.1%), hypertension (69.5%), waist circumference (57.7%), BMI (46.6%), vegetables (42.6%), smoking (39.3%), family history of T2DM (25.8%), and a sedentary lifestyle (24.1%). The difference in the AUC between the 2 models (i.e., the fourth model compared with the first model using the DeLong method) was not statistically significant (p= 0.588). Therefore, we removed a sedentary lifestyle from the fourth model and continued the modeling process with-out it.

### Fifth model

This model included 8 risk factors. These risk factors, in order of their importance, were waist circumference (100.0%), stress (77.2%), age (66.8%), BMI (63.5%), hypertension (59.9%), family history of T2DM (58.2%), smoking (41.8%), and vegetable consumption (27.1%). The AUC of the fifth and the first models did not show a significant difference (p= 0.217). Therefore, we ran the multilayer perceptron ANN model again without the risk variable of vegetable consumption.

### Sixth model

The sixth model included 7 risk factors: waist circumference (100.0%), age (78.5%), BMI (78.2%), hypertension (69.4%), stress (54.2%), smoking (49.3%), and family history of T2DM (37.2%), with the normalized importance rate of each risk factor shown in parentheses. The AUC of the sixth and the first models was not significantly different (p= 0.206). The sixth ANN model was run by 5 hidden-layer neurons. The final model contained 11 input neurons and 2 output neurons.

**Figure 2.**
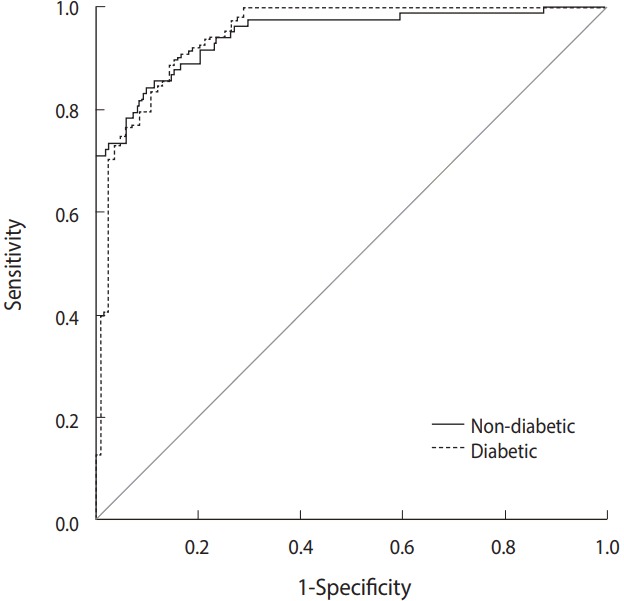
The area under the receiver operating characteristic curve for non-diabetic and diabetic subjects in the test and training groups based on the sixth model (final stage), containing waist circumference, age, body mass index, hypertension, stress, smoking, and family history of type 2 diabetes mellitus.

Since the AUC of the seventh model showed a significant difference compared to that of the first model (p= 0.024), the modeling process was considered to be complete. The final ANN model is shown in Figure 1. The risk factors selected in the sixth model were identified as the best predictors of T2DM. The goodness-offit indices of models 1 to 6 are shown in Table 3. As shown in Figure 2 and Table 3, model 6 showed suitable sensitivity, specificity, and AUC values.

## DISCUSSION

In this study, the diagnosis of T2DM was modeled using a multilayer perceptron ANN model. An ANN model based on a hidden layer can be used to delimit the relationships between the input variables and the output variable so that the best classification can be created. In contrast, linear models (e.g., multiple linear regression) cannot do this. The definition of such decision boundaries is possible with neural network models. Many studies [1,8,25] have been conducted on the prediction of diabetes mellitus using risk factors, but most of them have considered blood lipid parameters as risk factors, although they may not be applicable in largescale screening programs for T2DM. The demographic risk factors in this study, which do not require referral to the laboratory, can be more widely used than medical risk factors [1,8,25] in screening studies. This is potentially valuable because identifying people at high risk for T2DM is an important task in various communities.

Our results showed that waist circumference was very important for predicting T2DM, and was identified as the first predictor. Although the importance of waist circumference has not been confirmed in all previous studies, some studies, such as those conducted by Xu et al. [30] and Adhikary et al. [10], have reported an association between waist circumference and diabetes. Therefore, the results of this study are consistent with those of previous studies.

In the last ANN model, age was identified as the second most important factor, in accordance with previous studies [14,31]. Although age in the last ANN model ranked second, it should be noted that age had the first rank in the 5 prior ANN models. Therefore, it can be said that age is one of the strongest predictors for the diagnosis of T2DM.

Many studies [32-34] have shown that BMI may be related to T2DM, in accordance with our results. BMI was identified as the third strongest predictor for the diagnosis of T2DM in this study.

Hypertension was the fourth strongest predictor in our research, with an importance level of 69.4%. An association between T2DM and hypertension was also reported by Wise [32], Miyakawa et al. [33], and Walther et al. [34]. Adeyemo [1] used systolic and diastolic blood pressure to predict T2DM in his research. However, a single measurement of a patient’s blood pressure cannot be a reliable and valid risk factor for the diagnosis of T2DM because systolic and diastolic blood pressure readings are dependent on individual and environmental factors. We tried to measure the presence or absence of hypertension by 2 realistic questions. The first question was “Have you ever taken medication to control your blood pressure?”, and the second question was “Has a doctor ever told you that you have abnormal blood pressure?”

We found a few studies [17,18] that pointed out a relationship between stress and T2DM. We also measured participants’ stress by a simple question with a score of 0 to 10. The ANN results showed that stress levels were the fifth strongest predictor of a T2DM diagnosis. In other words, our study confirmed the results of previous studies [17,18,35]. Our results showed that stress was a more important predictor of T2DM than family history of diabetes or smoking status.

Akter et al. [36], in a systematic review and meta-analysis, showed a linear relationship between cigarette consumption and T2DM in the Japanese population, which is consistent with the outcome of our study. In this study, smoking was the sixth strongest predictor of T2DM.

The ANN model showed that the presence of diabetes among family members was a prognostic factor of T2DM. The importance of a family history of diabetes was also noted by van Zon et al. [13] and Adhikary et al. [10].

On the basis of 6 steps of modeling, we observed that risk factors such as waist, age, BMI, hypertension, stress, smoking, and family history of T2DM could play a valuable role in predicting T2DM. Therefore, we suggest that a tool should be developed based on these risk factors, in order to monitor those at high risk of T2DM and to identify undiagnosed cases of T2DM.

The risk factors in our study are cost-effective and simple to measure; virtually anyone can answer these questions in a few minutes and thereby assess his or her risk for T2DM. Another of the strengths of this study was the use of an ANN model to determine the importance of each of the risk factors. Determining the relative importance of each risk factor can be useful for health planning.

In conclusion, this study was a basic study for identifying people at high risk for T2DM. In this study, we examined demographic risk factors that do not require significant cost or time to measure in order to predict T2DM. Due to the sensitivity, specificity, and accuracy of the final model, it is suggested that these factors be used for assessing T2DM risk in screening tests.

This study had a few limitations. First, we would have liked to study more people, but due to a lack of funds, we could not increase the sample size. Second, in this study, only 1 question was used to measure stress levels, which may not be sufficiently precise. Since the participants did not want to answer a large number of questions, we had to measure stress level with a single question. Third, we measured the insufficient consumption of fruits and vegetables with 1 question. The reason for this was the reluctance of participants to respond to a large number of questions. Fourth, we evaluated walking in this study, although the results would have been more accurate if we had measured subjects’ physical activity more precisely.

## Figures and Tables

**Table 1.** Input and output variables for the neural network model

Status	Attributes	Levels	Code	Descriptions
Output	Diagnosis of T2DM (HbA1c)	<5.7%: normal	0	Dichotomous (%)
		≥5.7%: diabetic	1	
Input	Sex	Male	0	Dichotomous
		Female	1	
Input	Age	-	-	Numeric (yr)
Input	BMI^1^	-	-	Numeric (kg/m^2^)
Input	Hypertension^2^	Yes	1	Dichotomous
		No	0	
Input	Walking^3^	<30	0	Dichotomous (min/d)
		≥30	1	
Input	Sedentary time at workplace or home^4^	Sometimes	0	Dichotomous
		Often	1	
Input	Stress	-	-	Numeric (0-10)
Input	Fruit consumption^5^	Sometimes	0	Dichotomous
Input	Vegetables consumption^6^	Often	1	Dichotomous
Input	Family history of diabetes	Yes	1	Dichotomous
		No	0	
Input	Smoking (cigaretts, hookah)	Never	0	Categorical
		Former or current	1	
Input	Waist circumference	-	-	Numeric (cm)

T2DM, type 2 diabetes mellitus; HbA1c, hemoglobin A1c; BMI, body mass index.

1 BMI calculated as weight (kg)/height squared (m^2^).

2 Participants were considered to have hypertension if they took blood pressure medication.

3 Walking was collected as a dichotomous variable, walking less than 30 min/d was denoted by "0" and walking for more than 30 min/d was denoted as "1."

4 Sedentary time was defined in terms of the amount of time (hours) a person spent sitting at the office or at home; Sedentary time less than 5 hours was denoted as “sometimes,” and sedentary time for more than 5 hours was denoted as “always.”

5 Consumption of 0-1 servings of fruit per day was denoted as "sometimes," and consumption of ≥2 servings of fruit per day was denoted as "always."

6 Consumption of 0-1 cup of green vegetables per day was denoted as "sometimes," and consumption of ≥2 cups per day was denoted as "always."

**Table 2.** Risk factors used for univariate logistic regression

Variables	Normal (n=83)	T2DM (n=151)	OR (95% CI)
Sex	0.64 (0.20, 2.04)		
Male	13 (18.1)	59 (81.9)	
Female	70 (43.2)	92 (56.8)	
Age (yr)	36.54±10.70	53.25±11.20	1.24 (1.02, 1.53)^1^
BMI (kg/m^2^)	23.10±3.59	28.57±4.10	1.18 (0.98, 1.42)
Waist circumference (cm)	78.07±18.11	102.39±10.05	1.08 (1.01, 1.15)^1^
Stress (0-10)	5.55±2.25	5.44±2.69	1.42 (1.13, 1.79)^1^
Hypertension			4.52 (1.01, 12.27)^1^
No	80 (50.3)	79 (49.7)	
Yes	3 (4.0)	72 (96.0)	
Walking (min/d)			1.28 (0.41, 3,96)
<30	36 (27.3)	96 (72.7)	
≥30	47 (46.1)	55 (53.9)	
Sedentary time at workplace or home			6.06 (2.04, 8.04)^1^
Sometimes	50 (66.7)	25 (33.3)	
Often	33 (20.8)	126 (79.2)	
Fruit consumption			0.84 (0.164, 4.31)
Sometimes	9 (33.3)	18 (66.7)	
Often + always	74 (35.7)	133 (64.3)	
Vegetable consumption			0.07 (0.01, 0.44)^1^
Sometimes	4 (6.5)	58 (93.5)	
Often + always	79 (42.0)	93 (54.1)	
Family history of diabetes			2.94 (1.08, 7.83)^1^
No	73 (50.0)	73 (50.0)	
Yes	10 (11.4)	78 (86.6)	
Smoking (cigarettes, hookah)			4.26 (2.29, 7.93)^1^
Never	66 (47.8)	72 (52.2)	
Former + current	17 (7.7)	79 (82.3)	

Values are presented as number (%) or mean±standard deviation. T2DM, type 2 diabetes mellitus; OR, odds ratio; CI, confidence interval; BMI, body mass index.

1 ORs and 95% CIs were obtained by univariate logistic regression, and significant (p<0.2) risk factors.

**Table 3.** Results of multilayer perceptron neural network modeling

Models	Risk factors	Data set (test)	Sensitivity (%)	Specificity (%)	AUC	Accuracy (%)
1	Age, hypertension, waist circumference, BMI, sedentary lifestyle, smoking, vegetable consumption, family history of T2DM, stress, walking, fruit consumption, and sex	Training	96.2	76.7	0.947	89.2
			93.3	82.5	0.942	89.7
2	Age, hypertension, waist circumference, BMI, sedentary lifestyle, smoking, vegetable consumption, family history of T2DM, stress, walking, and fruit consumption	Training	94.0	79.6	0.920	90.9
			92.2	75.9	0.931	86.3
3	Age, hypertension, waist circumference, BMI, sedentary lifestyle, smoking, vegetable consumption, family history of T2DM, stress, and walking	Training	93.2	79.3	0.911	88.6
			95.1	80.0	0.920	89.8
4	Age, hypertension, waist circumference, BMI, sedentary lifestyle, smoking, vegetable consumption, family history of T2DM, and stress	Training	95.0	78.7	0.943	91.3
			96.1	63.6	0.945	86.3
5	Age, hypertension, waist circumference, BMI, smoking, vegetable consumption, family history of T2DM, and stress	Training	94.1	79.6	0.953	92.9
			95.2	82.5	0.963	96.9
6	Age, hypertension, waist circumference, BMI, smoking, family history of T2DM, and stress	Training	93.6	66.1	0.946	84.2
			95.2	88.9	0.953	92.8

AUC, area under the receiver operating characteristic curve; BMI, body mass index; T2DM, type 2 diabetes mellitus.
